# Searching, Structural Determination, and Diagnostic Performance Evaluation of Biomarker Molecules for Niemann–Pick Disease Type C Using Liquid Chromatography/Tandem Mass Spectrometry

**DOI:** 10.5702/massspectrometry.A0111

**Published:** 2022-12-23

**Authors:** Masamitsu Maekawa, Nariyasu Mano

**Affiliations:** 1 Department of Pharmaceutical Sciences, Tohoku University Hospital, Sendai, Japan

**Keywords:** biomarker, identification, quantification, LC/MS/MS, Niemann–Pick disease type C

## Abstract

Niemann–Pick disease type C (NPC) is an autosomal recessive disorder that is characterized by progressive neuronal degeneration. Patients with NPC have a wide age of onset and various clinical symptoms. Therefore, the discovery and diagnosis of NPC are very difficult. Conventional laboratory tests are complicated and time consuming. In this context, biomarker searches have recently been performed. Our research group has previously also investigated NPC biomarkers based on liquid chromatography/tandem mass spectrometry (LC/MS/MS) and related techniques. To identify biomarker candidates, nontargeted analysis with high-resolution MS and MS/MS scanning is commonly used. Structural speculation has been performed using LC/MS/MS fragmentation and chemical derivatization, while identification is performed by matching authentic standards and sample specimens. Diagnostic performance evaluation was performed using the validated LC/MS/MS method and analysis of samples from patients and control subjects. NPC biomarkers, which have been identified and evaluated in terms of performance, are various classes of lipid molecules. Oxysterols, cholenoic acids, and conjugates are cholesterol-derived molecules detected in the blood or urine. Plasma lyso-sphingolipids are biomarkers for both NPC and other lysosomal diseases. *N*-palmitoyl-*O*-phosphocholine-serine is a novel class of lipid biomarkers for NPC. This article reviews biomarkers for NPC and the analysis methods employed to that end.

## INTRODUCTION

Niemann–Pick disease is a group of diseases defined by Crocker in 1961.^1)^ Similar to Niemann–Pick disease, types A–D are present. Niemann–Pick disease types A and B (NPA/NPB) are caused by mutations in the *SMPD1* gene encoding acid sphingomyelinase.^2,3)^ The cause of Niemann–Pick disease type C (NPC) differs from that of *NPC1* and *NPC2* gene mutation.^4–7)^ Initially, a dysfunction of lysosomal cholesterol esterification was observed in NPC.^6)^ Subsequently, the lack of transport function of exogenous unesterified cholesterol in lysosomes was found to be the cause of NPC pathology.^8)^ NPC pathology is caused by two types of mutations, *NPC1* and *NPC2*.^9)^ The NPC1 protein expresses a lysosomal membrane transporter protein for unesterified cholesterol.^7,10)^ Another causative gene, *NPC2*, was identified as *HE1*.^11)^ The prevalence of NPC is estimated as 0.14–2.2 per 100,000 individuals,^5,12–18)^ wherein the ratio of to *NPC1*/*NPC2* in patients with NPC is approximately 95 : 5.^5)^

**Figure 1:**
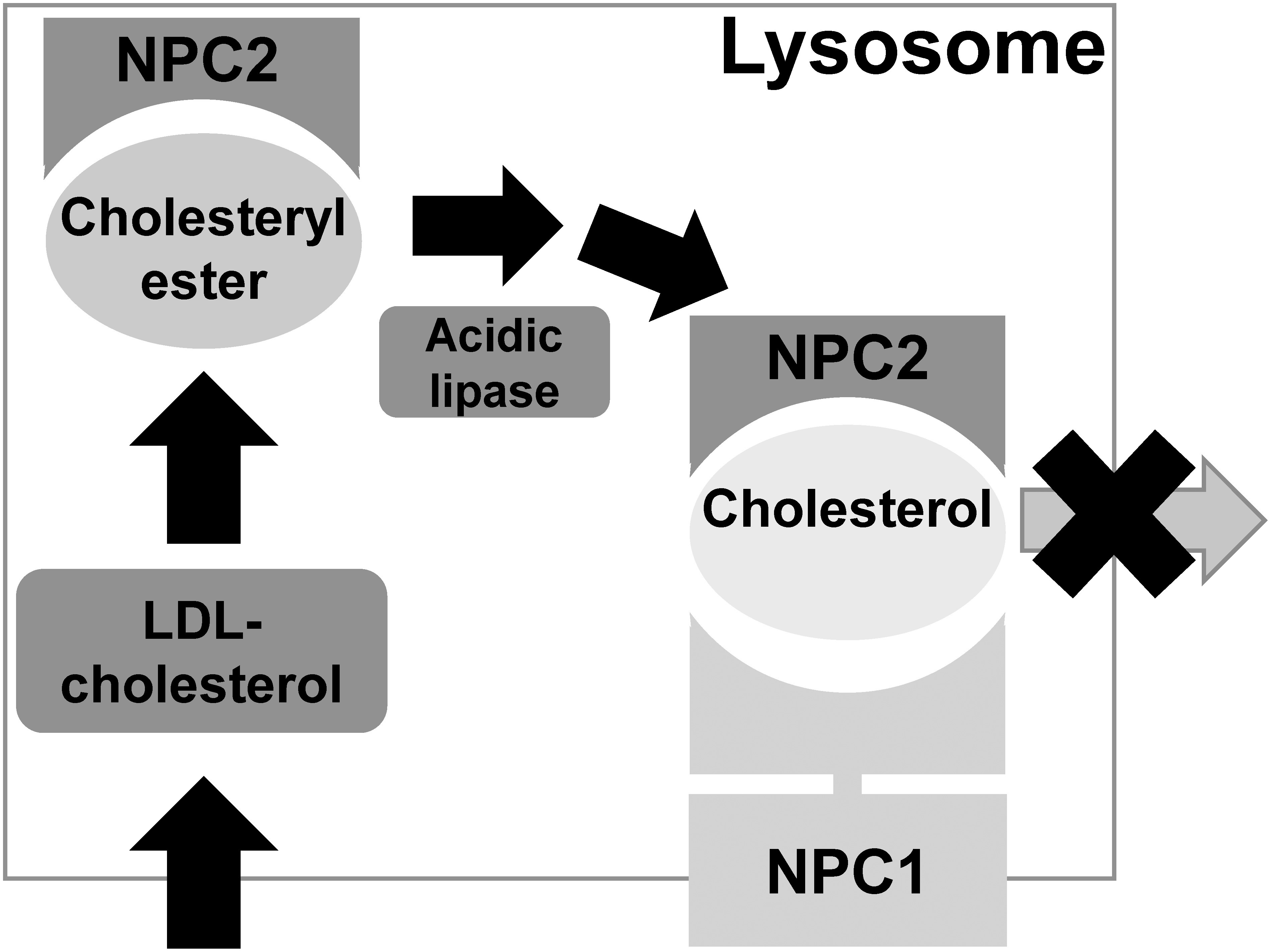
Fig. 1. Defect of cholesterol traffic in Niemann–Pick disease type C. In normal cells, cholesterol is taken up by lysosomes as LDL-cholesterol, hydrolyzed to the unesterified form, and transported out of the lysosomes. However, in the cells of individuals with NPC, the transport of unesterified cholesterol is defective due to the functional lack of NPC1 or NPC2 proteins, resulting in the accumulation of unesterified cholesterol.

In normal cells, cholesterol is taken up by lysosomes as LDL-cholesterol, hydrolyzed to the unesterified form, and transported out of lysosomes. The NPC2 protein binds and transports unesterified cholesterol, after which it is exported by the NPC1 protein.^19)^ However, in NPC cells, the transport of unesterified cholesterol is impaired due to the functional lack of NPC1 or NPC2 proteins, resulting in the accumulation of unesterified cholesterol^19)^ (Fig. 1).

NPC patients exhibit pathological symptoms in the central nervous system, peripheral nerves, and systemic organs. By the age of onset, they are generally classified into five types: (1) perinatal (birth–2 months), (2) early infantile (2 months–2 years), (3) late childhood (2–6 years), (4) young (6–15 years), and (5) adolescent/adulthood (≥15 years).^5,8,9)^ In general, the degree of lipid accumulation is high in young-onset cases, including cholestasis in the neonatal period.^9)^ Cataplexy, acute seizures, and narcolepsy are typically observed.^9)^ In addition, cerebellar ataxia, dystonia, dysarthria, and epilepsy have also been observed in patients with NPC,^8)^ and psychiatric symptoms similar to those of schizophrenia are commonly observed.^20)^ In a study regarding psychiatric symptoms, the genome sequencing analysis of 250 patients with schizophrenia identified three patients with NPC.^21)^

Generally, two gold standard methods have been used as conventional diagnostic tests for NPC, namely filipin staining and DNA mutation analysis.^9)^ Filipin staining is a pathological method based on staining accumulated cholesterol in the cultured cells of biopsy samples from NPC patients.^6,22–24)^ This method requires highly invasive biopsy, a long cell culturing time, and complex staining procedures. In some cases, false-negative results with weak staining are found.^22)^ In DNA mutation analysis, mutations in both *NPC1* and *NPC2* are analyzed. Conventionally, PCR^25)^ and denaturing HPLC^26)^ are used. In recent years, next-generation sequencing has also been employed.^27–31)^ Many mutations have been reported to date, and novel mutations continue to be characterized.^32)^ However, a significant disadvantage of these methods is their high running cost.

Miglustat is the only therapeutic drug approved for the treatment of NPC.^33)^ This drug inhibits central nervous system progression and a better prognosis in the case of early treatment.^34)^ Improvements in neurological symptoms have also been observed.^35)^ 2-Hydroxypropyl-β-cyclodextrin (HPBCD), a cyclodextrin derivative with elimination activity against cellular accumulated cholesterol, has also been reported to be an effective therapeutic agent for NPC.^36,37)^ A recent clinical trial showed effective results for HPBCD in patients with NPC.^38)^ In Japan, the intravenous and intracerebroventricular administration of HPBCD have been performed.^39,40)^ At present, a variety of agents are being investigated for improved efficacy in the treatment of NPC.^41,42)^

## BIOMARKERS FOR NPC

**Figure 2:**
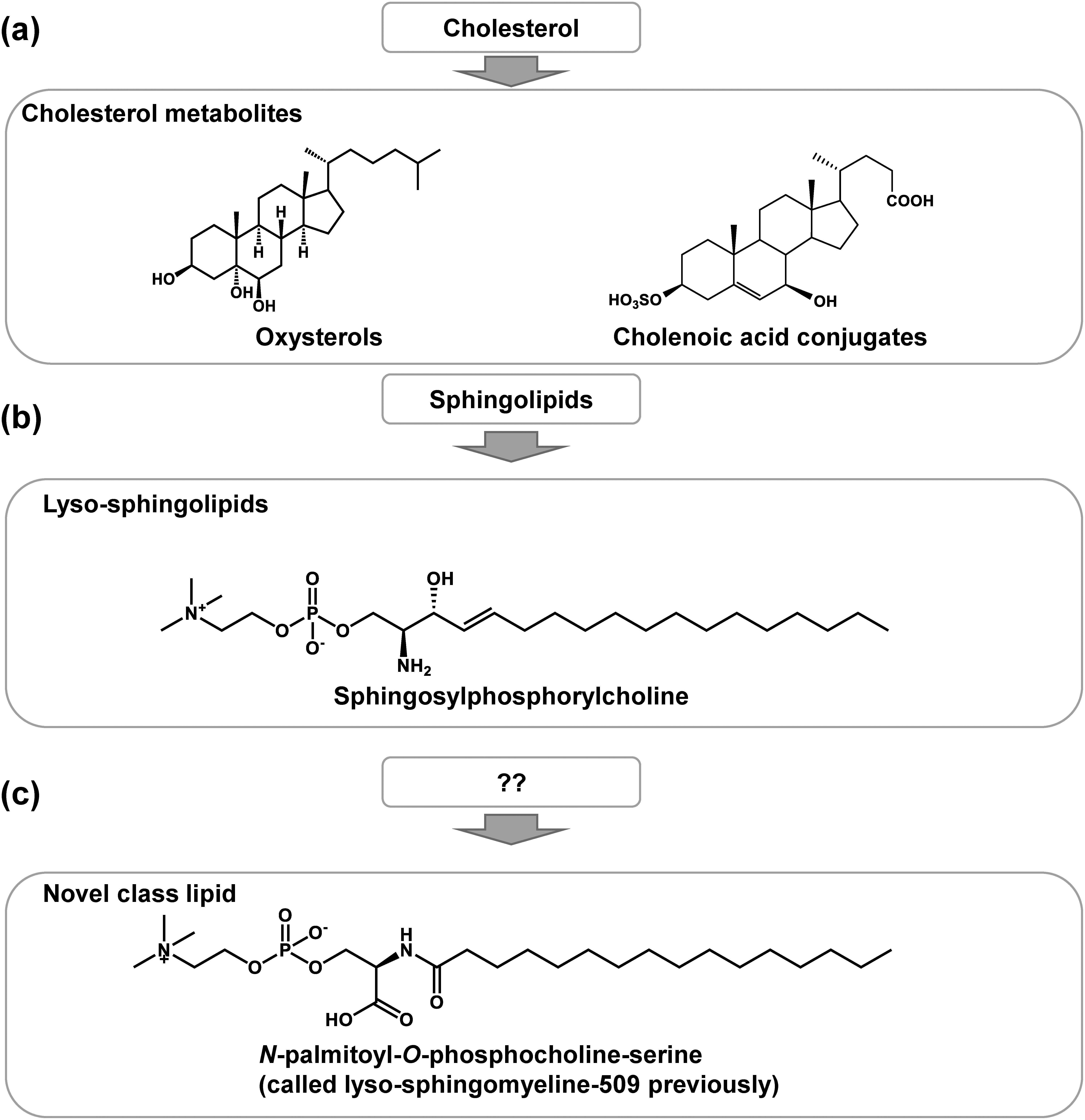
Fig. 2. Recently reported lipid metabolites as NPC biomarkers: (a) cholesterol metabolites: oxysterols, cholenoic acids, and the conjugates; (b) lyso-sphingolipids: sphingosylphosphorylcholine; (c) novel class lipid: *N*-palmitoyl-*O*-phosphocholine-serine (previously known as lyso-sphingomyeline-509).

**Figure 3:**
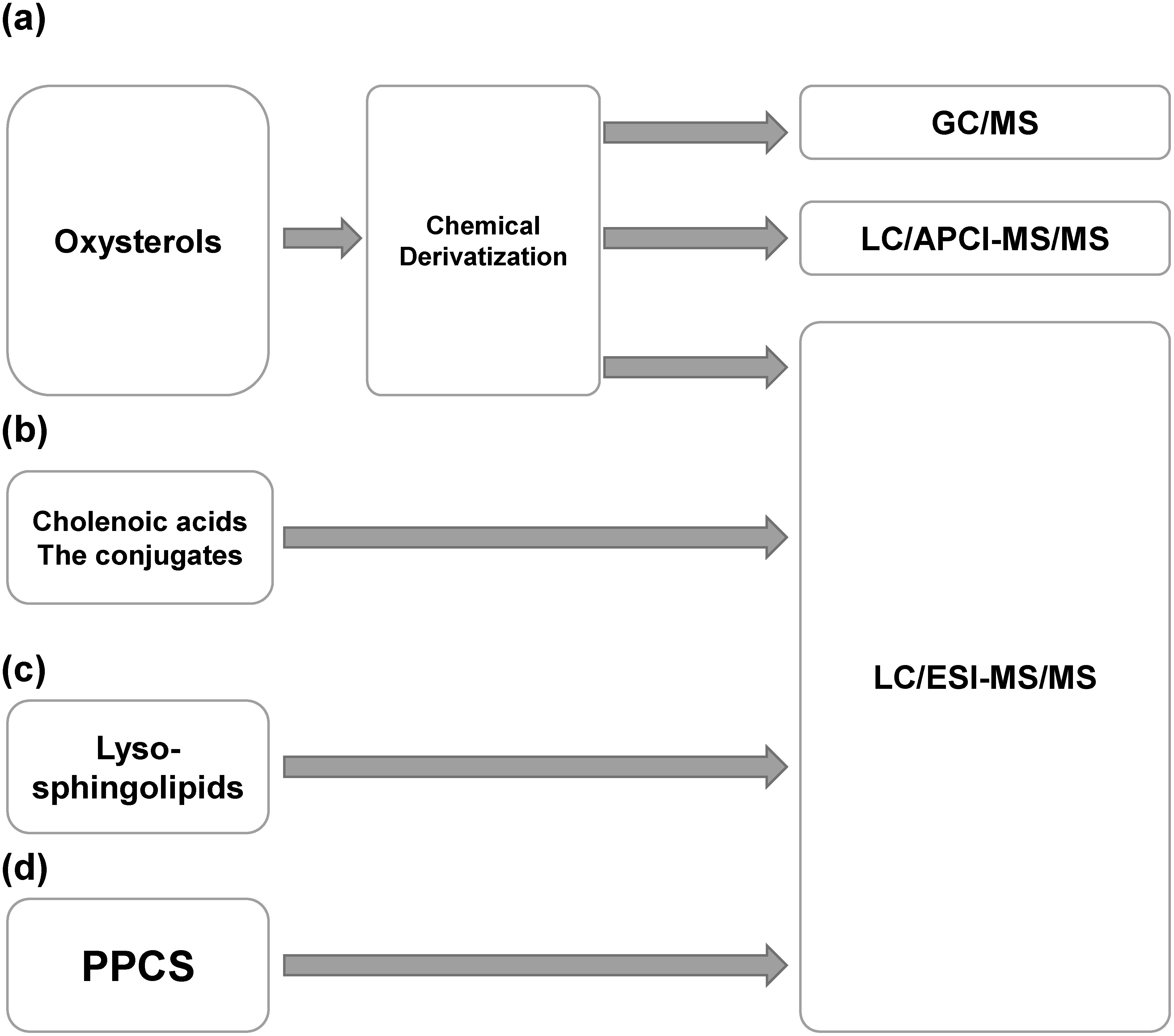
Fig. 3. NPC biomarkers and typical analytical methods: (a) oxysterols: they are firstly derivatized and analyzed with various methods (GC/MS or LC/APCI-MS/MS or LC/ESI-MS/MS); (b) cholenoic acids and conjugates; (c) lyso-sphingolipids; (d) PPCS. Abbreviations: APCI, atmospheric pressure chemical ionization; ESI, electrospray ionization; GC, gas chromatography; MS, mass spectrometry; MS/MS, tandem mass spectrometry; PPCS, *N*-palmitoyl-*O*-phosphocholine-serine.

Because of the importance of early treatment and the shortcomings of conventional diagnostic methods for NPC,^5,23)^ biomarkers and chemical diagnoses have received significant attention. In fact, many biomarkers of NPC have been reported in the last two decades. All of these biomarkers were identified and quantified using mass spectrometry (MS) and related techniques. Our own research group has reported biomarkers for NPC using mass spectrometric analysis.^43–46)^ Here, we provide an overview of known biomarkers for NPC (Fig. 2) and the mass spectrometric analysis methods employed for their identification (Fig. 3).

## PLASMA OXYSTEROLS AND ANALYSIS METHODS IN BLOOD

The first reported biomarkers for NPC were oxysterols,^47,48)^ which are oxidized cholesterol molecules. Generally, oxysterols are both enzymatically and non-enzymatically produced.^49,50)^ Oxysterols are non-enzymatically produced as biomarkers for NPC.^47)^ An increase in oxysterols in NPC cells was first observed in a rat models.^51)^ These biomarkers are increased by oxidative stress in NPC pathology.^52,53)^ In NPC, free radicals are increased,^54)^ and antioxidant capacity in NPC pathology is reduced.^55)^ 7β-Hydroxycholesterol (OHC), 7-ketocholesterol (7-KC), and 5β-cholestan-3β,5α,6β-triol (C-triol) are non-enzymatically produced oxysterols as NPC biomarkers. 4β-OHC, 7α-OHC, and 25-OHC are produced *via* both enzymatic and non-enzymatic pathways. 24*S*-OHC is used as a biomarker for neurological pathophysiology.^38,56,57)^

Various methods have been used to analyze oxysterols. In the first report of an animal model^51)^ and human specimen plasma,^47)^ gas chromatography/mass spectrometry (GC/MS) was used. Tint *et al.* performed pretreatment with liquid–liquid extraction and induced derivatization with trimethylsilyl ether. Among the various organs analyzed, liver 7β-OHC was found to increase significantly (6-fold) in NPC mice compared to control mice.^51)^ Porter *et al.* also used GC/MS to analyze oxysterols in plasma.^47)^ Saponification was performed to hydrolyze oxysterol esters after extraction using the Bligh and Dyer method. After adding an internal standard (IS), the analytes and IS were derivatized to their respective trimethylsilyl ethers, and an aliquot was used for GC/MS. A run time of 40 min allowed for identification and quantification. Because oxysterols have many structural isomers, chromatographic separation is essential. Porter *et al.* performed a preliminary examination of 22 oxysterol analyses of 10 patients with NPC and compared them with those reported in the literature. As a result of the preliminary comparison, an increase in C-triol and 7-KC and a decrease in 24S-OHC were observed.^47)^ Next, they analyzed a larger number of specimens. Plasma samples were collected from 25 NPC1 patients, 23 heterozygotes of NPC1 subjects, and 25 control subjects and analyzed for C-triol, 7-KC, and 24-OHC in the plasma samples. As a result, C-triol and 7-KC increased significantly in NPC1 patients compared to healthy controls.^47)^ In contrast, 24S-OHC was lower in NPC patients than in the healthy controls. In terms of stability, C-triol provided a 25% increase after a processing delay at room temperature. Diurnal variations of up to 23% were observed, and the two oxysterols 7-KC and C-triol were significantly correlated. In addition, the plasma concentrations and NPC disease onset age were found to be negatively correlated. As a result of receiver operative curve (ROC) analysis for the evaluation of diagnostic performance for NPC, the areas under the curve (AUC) of C-triol and 7-KC were 1.0 and 0.9984. In conclusion, C-triol and 7-KC were found to be excellent plasma biomarkers for NPC.^47)^

In 2011, Jiang *et al.* reported a rapid LC/MS/MS method for oxysterols for NPC diagnostic screening.^48)^ Compared to a previous report,^47)^ the chromatographic running time was shortened to 8.5 min, and high-speed analysis was performed using a C18 column and gradient elution. However, derivatization was required to increase the ionization and fragmentation efficiency of the MS/MS process for oxysterols. *N*,*N*-dimethylglycine (DMG) was used as the derivatization reagent. In the derivatization reaction, 4-(dimethylamino) pyridine and *N*-(3-dimethylaminopropyl)-*N*′-ethylcarbodiimide hydrochloride were used as the base and condensing agents, respectively. In the derivatization reaction, because 7-KC has a hydroxy group, it reacted with DMG in a 1 : 1 ratio. However, because C-triol has multiple hydroxy groups, it reacts with two DMG other than the 5α-hydroxy group. The authors used atmospheric pressure chemical ionization (APCI) as the ionization mode. As selected reaction monitoring (SRM) transitions, singly charged protonated DMG-derivatized molecules were selected as precursor ions, and DMG-derived ions at *m*/*z* 104 were selected as product ions. This study also investigated the matrix effect. When developing and performing biomarker analysis using LC/MS/MS, the sample matrix contains the analyte, even in healthy controls. Therefore, the matrix effect, which differs in MS intensity between the real matrix and solvent, should be investigated. In this report, the matrix effect was not observed in LC/MS/MS.^48)^ For diagnostic performance evaluation, plasma concentrations of 7-KC and C-triol were analyzed and compared with those in healthy controls and patients with NPC. The diagnostic performance was similar to that of a previous report.^47)^

Other chemical derivations have been used for the analysis of oxysterols using LC/MS/MS.^58–61)^ Picolinic acid is the most popular reagent for chemical derivatization and reacts with hydroxy groups. The picolinic derivatives were ionized as pronated molecules ([M+H]^+^),^60,62,63)^ acetonitrile adduct ions ([M+CH_3_CN]^+^),^60,62)^ sodium adduct ions ([M+Na]^+^),^59,61,64)^ and sodium and acetonitrile adduct ions ([M+Na+CH_3_CN]^+^).^58)^ They were affected by the mobile phase, LC system, and MS/MS conditions. Ionized picolinic acid or dissociated picolinic acid ions were detected as product ions.

Enzyme-assisted derivatization has also been reported a method for the analysis of oxysterol.^65)^ In this method, the partial structures of 3β-hydroxy-5-cholestene are converted to 4-cholesten-3-one by cholesterol oxidase and derivatized using Girard’s P reagent. The derivatives provided a neutral loss of 79 Da as the characteristic product ions.

Lin *et al.* analyzed 7-KC using LC/MS/MS without derivatization in electrospray ionization (ESI) mode and an ammonium adduct ion as a precursor ion.^66)^

## ANALYSIS OF ABNORMAL CHOLANOIC ACIDS IN BLOOD AND URINE USING LC/MS/MS

Abnormal cholenoic acids and their conjugates are NPC biomarkers that are classified as other cholesterol metabolites. In inherited metabolic disorders involving cholesterol metabolism, various bile acids,^67–73)^ abnormal cholenoic acids,^74–77)^ and their conjugates^74–77)^ have been detected in the urine or blood.

**Figure 4:**
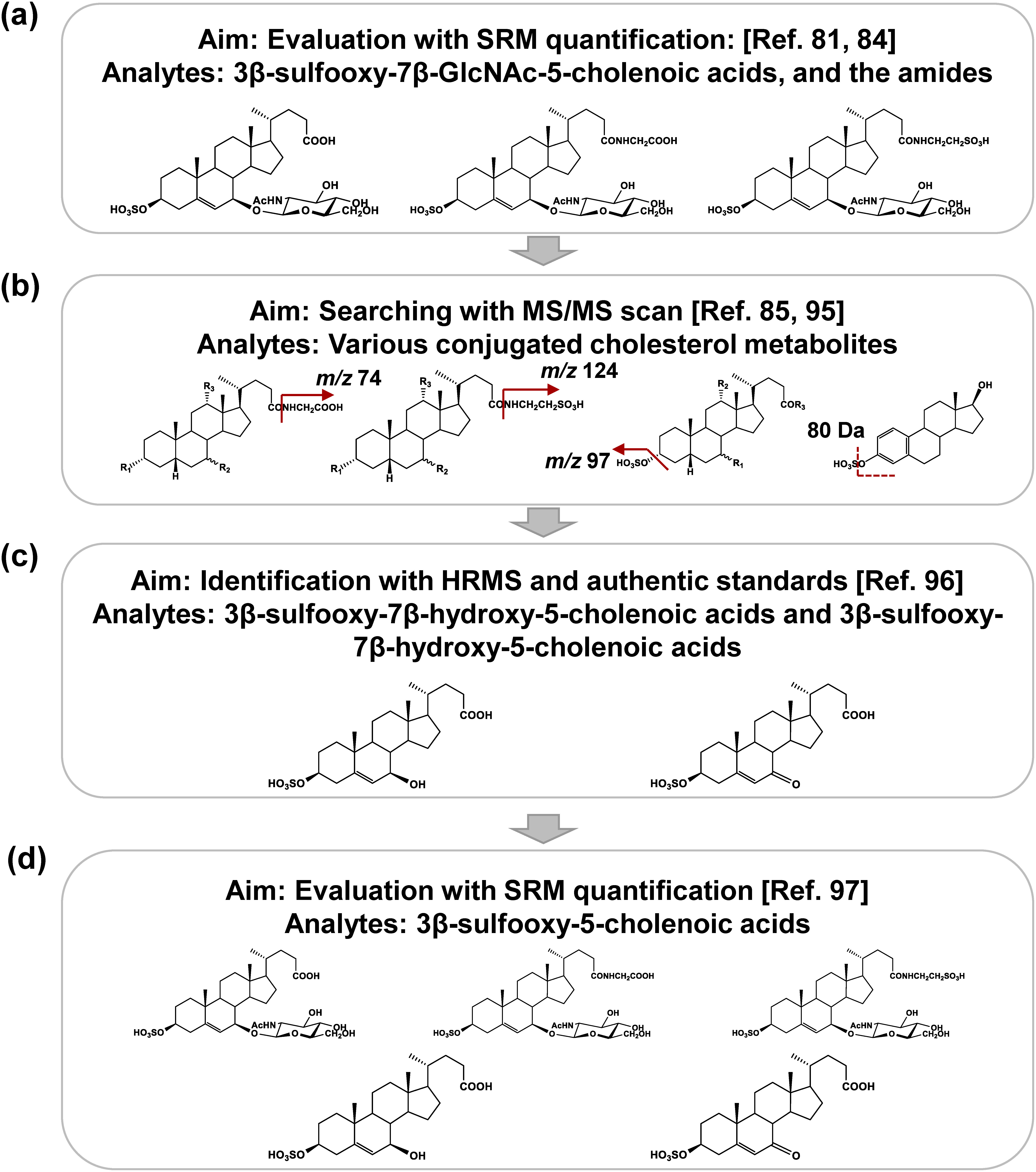
Fig. 4. Analytical methods for the evaluation of urinary cholenoic acid conjugates as NPC biomarkers: (a) 3β-sulfooxy-7β-GlcNAc-5-cholenoic acid and the amides; (b) focused metabolome analysis; (c) 3β-sulfooxy-7β-hydroxy-5-cholenoic acid and 3β-sulfooxy-7-oxo-5-cholenoic acid; (d) 3β-sulfooxy-7β-GlcNAc-5-cholenoic acid and the amides, 3β-sulfooxy-7β-hydroxy-5-cholenoic acid, and 3β-sulfooxy-7-oxo-5-cholenoic acid.

Studies on the use of cholenoic acids as NPC biomarkers are summarized in Fig. 4. In 2001, Alvelius *et al.* reported multiple conjugated cholesterol metabolites with 3β-hydroxy-5-cholen-skeletons in the urine of individuals with NPC.^78)^ The authors used this enzyme for deconjugation and GC-MS analysis for skeleton determination. The results of their nano-ESI-MS analysis suggested that 3β,7β-dihydroxy-5-cholenoic acid conjugates with sulfuric acid and *N*-acetylglucosamine (GlcNAc). The authors only reported one case of an NPC patient, and continuous reports for the evaluation of diagnostic performance based on quantitative analysis have not been reported since.

**Figure 5:**
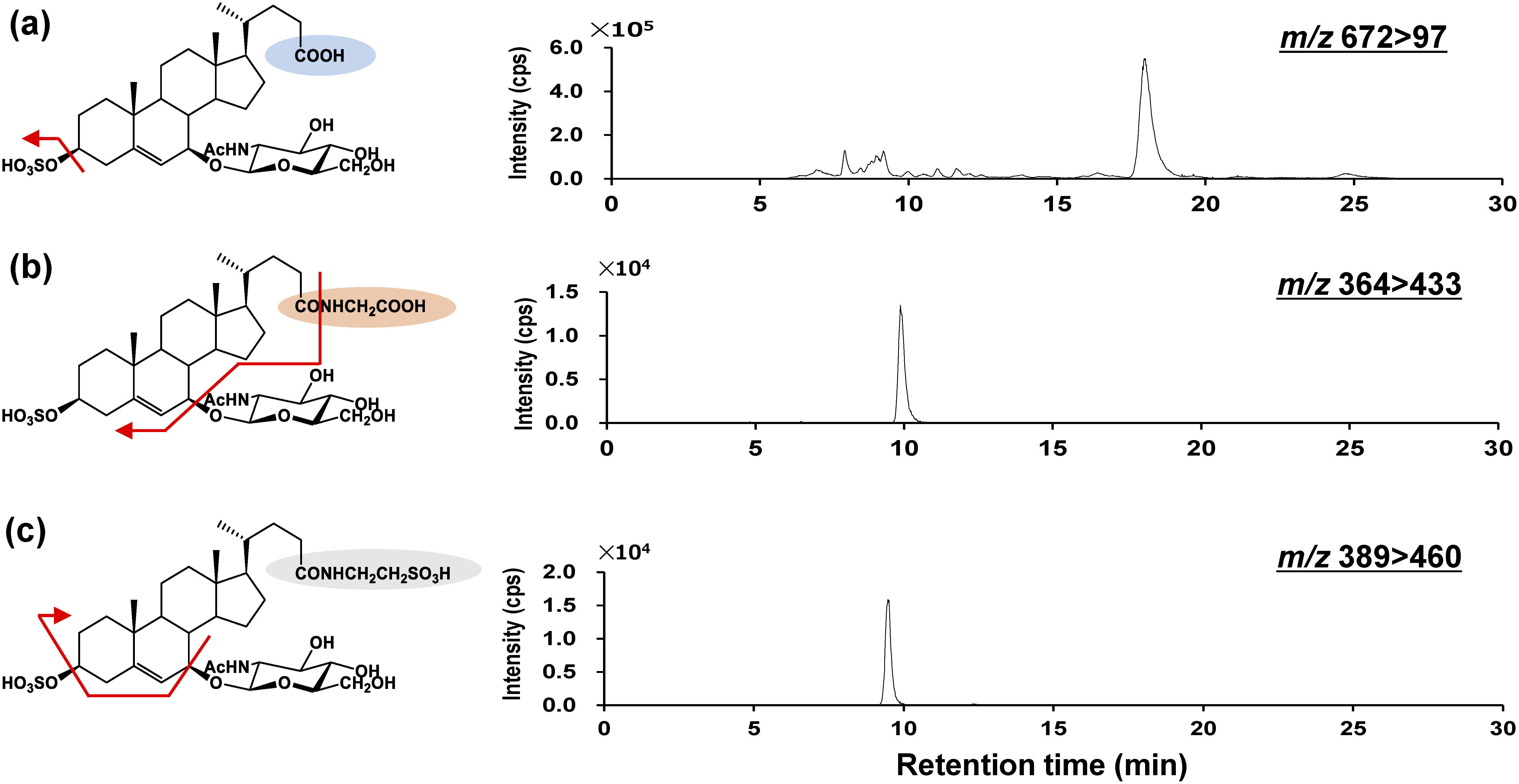
Fig. 5. SRM chromatograms of cholesterol metabolites in urine. (a) 3β-sulfooxy-7β-GlcNAc-5-cholenoic acid; (b) glycine conjugated 3β-sulfooxy-7β-GlcNAc-5-cholenoic acid; (c) taurine conjugated 3β-sulfooxy-7β-GlcNAc-5-cholenoic acid. Abbreviations: GlcNAc, *N*-acetylglucosamine; SRM, selected reaction monitoring.

**Figure 6:**
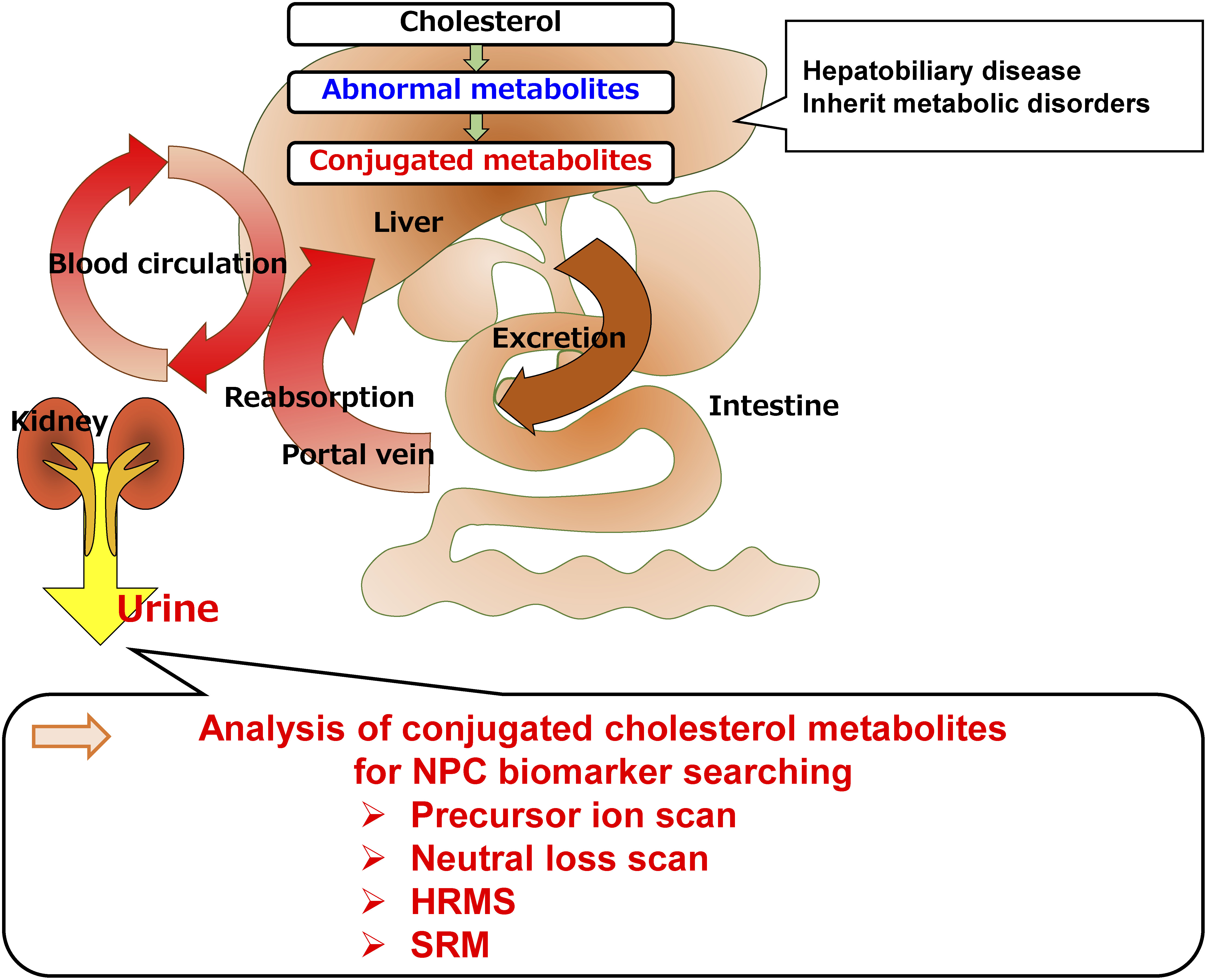
Fig. 6. Changes in cholesterol metabolism in body fluids in various diseases and analysis for urinary cholesterol metabolites.

In 2006, Iida *et al.* synthesized 3β,7β-dihydroxy-5-cholenoic acid using chemical methods.^79)^ In 2009, Kakiyama *et al.* synthesized 3β-sulfooxy-7β-OH-24-*nor*-5-cholenoic acid as a possible IS.^80)^ Using authentic standards and IS, in 2013, we developed an LC/MS/MS analysis method for the determination of 3β-sulfooxy-7β-GlcNAc-5-cholenoic acid and its glycine and taurine conjugates (SNAG-Δ^5^-CA, SNAG-Δ^5^-CG, and SNAG-Δ^5^-CT) using LC/MS/MS^81)^ (Fig. 5). A column switching system was used for pretreatment and chromatographic separation. Shim-pack MAYI-C8 (Shimadzu GLC, Kyoto, Japan), which is a methylcellulose-immobilized reversed-phase column for the elimination of protein in the sample matrix,^82,83)^ was used as a trapping column. A C18 column was used as the analytical column with a mixture of 20 mmol/L ammonium acetate buffer (pH 5.5) : methanol (1 : 1, v/v) as the mobile phase. For urinalysis, 33 ng/mL of the IS solution was added at a 1 : 1 (v/v) ratio to the urine, and 50 μL of the sample aliquot was injected for the analysis. In MS, SNAG-Δ^5^-CA and IS were ionized as [M−H]^−^, and SNAG-Δ^5^-CG and SNAG-Δ^5^-CT were ionized as [M−2H]^2−^, respectively.^81)^ In MS/MS, all compounds provided HSO_4_^−^ at *m*/*z* 97; however, for SNAG-Δ^5^-CG and SNAG-Δ^5^-CT, ions with neutral loss of various conjugated groups were detected because the *S*/*N* was higher than *m*/*z* 97. Finally, the SRM conditions were set as *m*/*z* 672>97, 364>433, 389>460, and 455>97 for SNAG-Δ^5^-CA, SNAG-Δ^5^-CG, SNAG-Δ^5^-CT, and the IS, respectively. This analytical method was validated and applied to urinalysis. Only two specimens from patients with NPC were analyzed in this study, showing higher metabolite concentrations than those of healthy subjects and controls with other metabolic abnormalities.^81)^ Urine samples were collected from healthy subjects and patients with NPC, cerebrotendinous xanthomatosis, abetalipoproteinemia, glycogen storage disease type 1, and glycogen storage disease type 2.^84)^ In a comparison of the urinary concentrations NPC patients and healthy controls, the three metabolites (SNAG-Δ^5^-CA, SNAG-Δ^5^-CG, and SNAG-Δ^5^-CT) in the urine of patients with NPC were significantly higher than those in the controls. Thus, these urinary metabolites showed good diagnostic performance, with differences in the average concentrations of approximately 360–570 times between patients with NPC and the healthy subjects. In the evaluation of diagnostic performance with ROC analysis, the AUC of SNAG-Δ^5^-CA, SNAG-Δ^5^-CG, and SNAG-Δ^5^-CT were over 0.95. The sensitivities of SNAG-Δ^5^-CA, SNAG-Δ^5^-CG, and SNAG-Δ^5^-CT were >95%, with specificities of 100%, 100%, and 92.3%, respectively. In other metabolic abnormalities, the urinary concentrations of SNAG-Δ^5^-CA, SNAG-Δ^5^-CG, and SNAG-Δ^5^-CT were lower than those in NPC. Therefore, SNAG-Δ^5^-CA, SNAG-Δ^5^-CG, and SNAG-Δ^5^-CT were found to have high diagnostic performance for NPC; however, some patients with NPC produced false-negative results.^84)^ We aimed to perform a novel metabolome analysis focused on searching for conjugated cholesterol metabolites to identify more accurate diagnostic candidates^85)^ (Fig. 6). In general, conjugated cholesterol metabolites containing bile acids can be analyzed with high sensitivity using LC/ESI-MS/MS.^86–91)^ Abnormally conjugated cholesterol metabolites are found in many cholesterol-related inherited metabolic and hepatobiliary diseases.^74,76,77,92–94)^ Therefore, the breakdown of cholesterol homeostasis causes abnormal cholesterol metabolism, with abnormal metabolites being potential biomarkers in body fluids.

**Figure 7:**
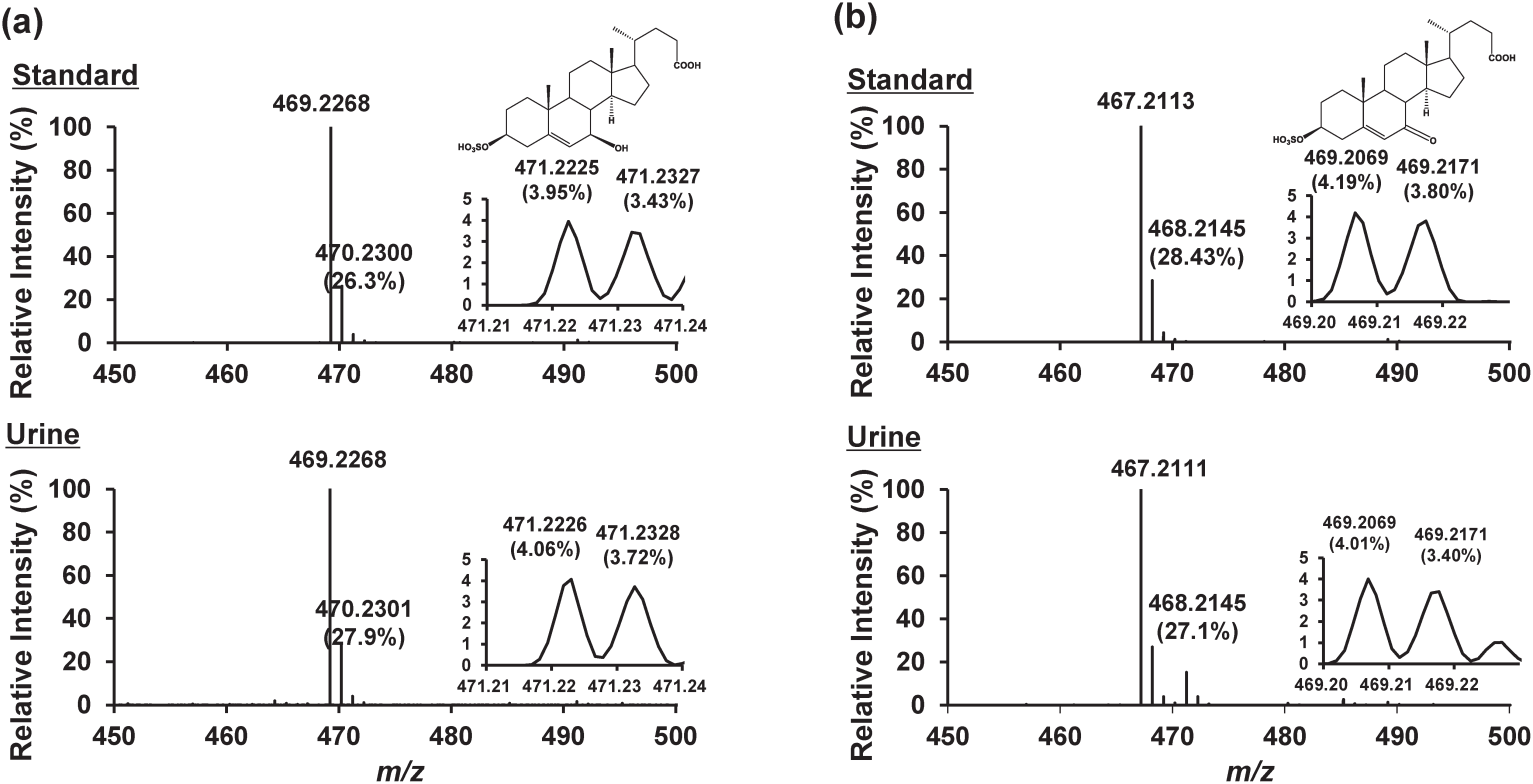
Fig. 7. High resolution mass spectra of standards and urine sample of a patient with NPC: (a) 3β-sulfooxy-7β-hydroxy-5-cholenoic acid; (b) 3β-sulfooxy-7-oxo-5-cholenoic acid.

We investigated the MS/MS fragmentation behaviors of conjugated cholesterol metabolites^95)^ and the collision-induced dissociation (CID) patterns of these metabolites. Every conjugated metabolite provided characteristic product ions and neutral losses for each conjugated group. For example, glycine-conjugated metabolites produce NH_2_CH_2_COO^−^ at *m*/*z* 74. The taurine-conjugated metabolites produced SO_3_^−^ at *m*/*z* 80, CH_2_CHSO_3_^−^ at *m*/*z* 107, and NH_2_CH_2_CH_2_SO_3_^−^ at *m*/*z* 124. Acyl-type 24-glucuronidated metabolites produced a loss of a 162 Da ion and glucuronic acid-derived ion at *m*/*z* 161 and 113. A sulfate ester through phenolic hydroxy group produced a neutral loss ion of 80 Da and bile acid sulfates produced HSO_4_^−^ at *m*/*z* 97 and various neutral loss ions. Based on these results, a focused metabolome analysis method for conjugates based on CID patterns was developed.^85)^ This method has been used in both precursor ion and neutral loss scans. First, a standard mixture of 80 compounds was prepared and used for analysis. Precursor ion scan analyses were performed for the conjugates that produced the characteristic product ions. Neutral loss scan analyses were performed for the conjugates that produced ions with constant neutral loss. We succeeded in the comprehensive analysis of targeted molecules with functional groups in standard mixture analysis. This method was applied to urine samples. Although a few peaks were observed in the urine of healthy subjects, many peaks were observed in the urine of patients with NPC and a 3β-hydroxy-Δ^5^-C_27_-steroid dehydrogenase deficiency. As a result, 140 peaks were observed in the urine of NPC patients. Intense peaks were observed for SNAG-Δ^5^-CA, SNAG-Δ^5^-CG, and SNAG-Δ^5^-CT. As a result, three intense characteristic peaks were detected in the urine of the patients with NPC (*m*/*z* 469.6, retention time 21.0 min; *m*/*z* 467.6, retention time 23.4 min; and *m*/*z* 453.5, retention time 16.1 min, respectively). Next, we analyzed them using high-resolution mass spectrometry (HRMS), and speculated their chemical formula. As a result, the peak at *m*/*z* 453.5 was not speculated to be a cholesterol metabolite. In contrast, the peaks at *m*/*z* 469.6 and the peak at *m*/*z* 467.6 were proposed to be C24H38O7S and C24H36O7S, respectively, based on their accurate masses. Considering their LC retention times and the proposed formulas, the structures were suggested to be 3β,7β-dihydroxy-5-cholenoic acid 3-sulfate (S7B-Δ^5^-CA) and 3β-hydroxy-7-oxo-5-cholenoic acid 3-sulfate (S7O-Δ^5^-CA), respectively. Next, the speculated compounds were chemically synthesized and analyzed under the same conditions as in the urine of NPC patients. The LC/MS/MS analytical results matched the authentic standards and urine of patients with NPC (Fig. 7). In conclusion, the two urine peaks were successfully identified as S7B-Δ^5^-CA and S7O-Δ^5^-CA and represented as new biomarker candidates for NPC.^96)^

**Figure 8:**
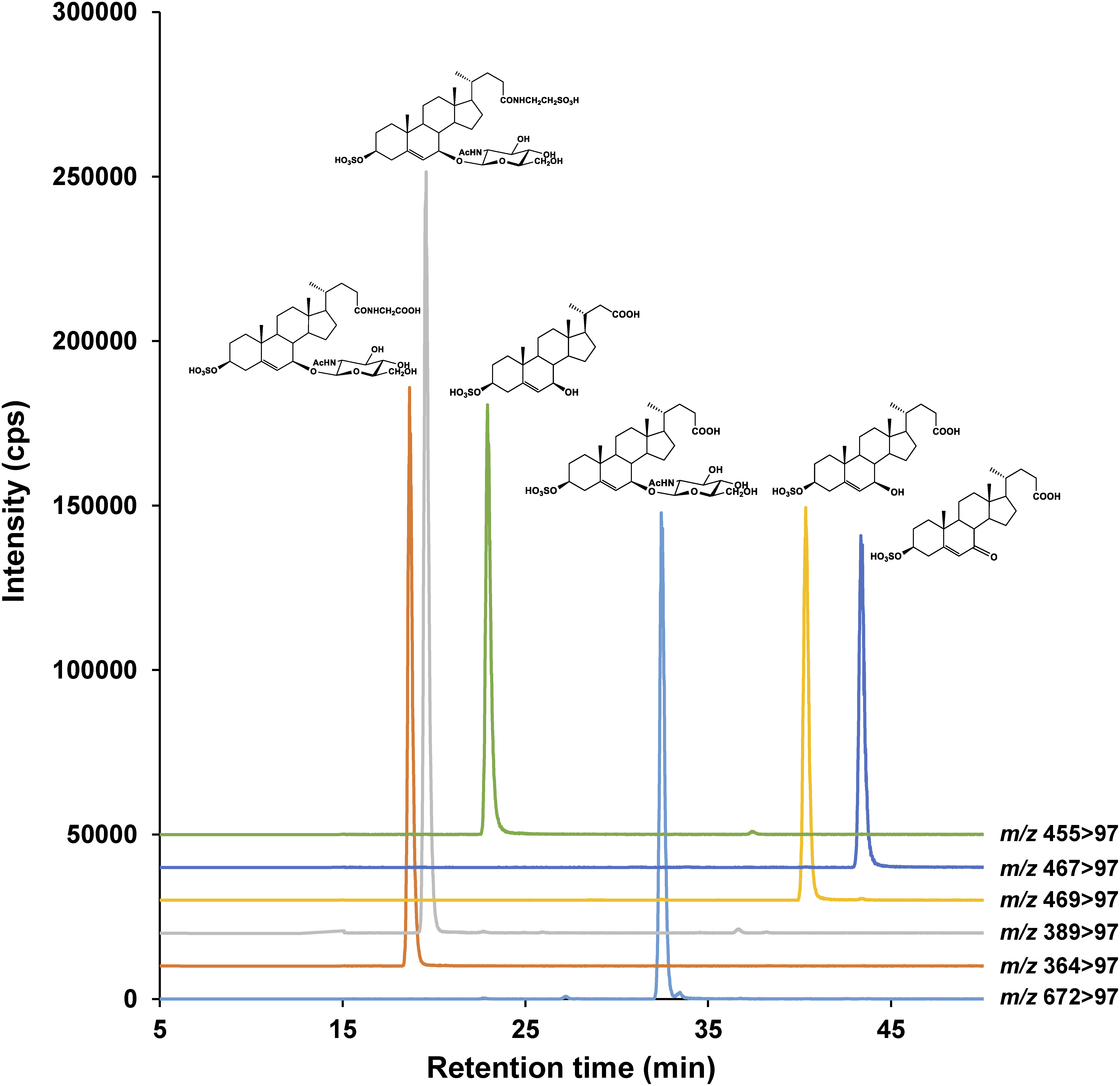
Fig. 8. SRM chromatograms of urinary cholesterol metabolites. 3β-sulfooxy-7β-GlcNAc-5-cholenoic acid, glycine-conjugated 3β-sulfooxy-7β-GlcNAc-5-cholenoic acid, taurine-conjugated 3β-sulfooxy-7β-GlcNAc-5-cholenoic acid, 3β-sulfooxy-7β-hydroxy-5-cholenoic acid, 3β-sulfooxy-7-oxo-5-cholenoic acid, and 3β-sulfooxy-7β-hydroxy-24-*nor*-5-cholenoic acid (internal standard) were detected at *m*/*z* 672>97, 364>97, 389>97, 469>97, 467>97, and 455>97, respectively. Abbreviations: SRM, selected reaction monitoring.

Next, a simultaneous LC/MS/MS quantitative analysis method for SNAG-Δ^5^-CA, SNAG-Δ^5^-CG, SNAG-Δ^5^-CT, S7B-Δ^5^-CA, and S7O-Δ^5^-CA in urine was developed and its diagnostic performance was evaluated.^97)^ S7B-Δ^5^-CA and S7O-Δ^5^-CA were detected as [M−H]^−^ and provided only HSO_4_^−^ at *m*/*z* 97 as an intense product ion. In the MS/MS analysis of urine, because the structures of S7B-Δ^5^-CA and S7O-Δ^5^-CA are simpler than those of SNAG-Δ^5^-CA, SNAG-Δ^5^-CG, and SNAG-Δ^5^-CT, many urinary interfering peaks were detected. Therefore, complete chromatographic separation is required for accurate quantification. We succeeded in optimizing the LC conditions with an LC 60 min gradient elution (Fig. 8). This method was not affected by the matrix effect in urine, and the calibration curves had satisfactory ranges. Analytical method validation was achieved with guidance from previous studies.^98)^ To evaluate the diagnostic performance, urine samples from healthy subjects, patients with NPC, and patients with other control diseases (cerebrotendinous xanthomatosis, Gaucher disease, Hunter disease, and Smith-Lemli-Opitz syndrome) were analyzed. The concentrations of the five metabolites in the urine of patients with NPC were significantly higher than those in the healthy subjects. In addition, in the ROC analysis, all metabolites showed excellent diagnostic performance, with AUC values of >0.9. In particular, S7B-Δ^5^-CA showed an AUC of 1.0 and 100% sensitivity and specificity, respectively. Urine is an alternative specimen to blood. Thus, these molecules may be useful urinary biomarkers for NPC diagnosis.

Mazzacuva *et al.* and Jiang *et al.* reported that some non-sulfate conjugated cholenoic acids in the blood may be useful for the diagnosis of NPC.^99,100)^ Both groups performed a comprehensive analysis to identify peak candidates in the blood, and both groups finally identified two metabolites. One of these, 3β-hydroxy-7β-GlcNAc-5-cholenoic acid, is a sulfated metabolite of SNAG-Δ^5^-CA. GlcNAc conjugation is catalyzed by UGT3A1,^101)^ and the conjugates are easily excreted in the urine.^102,103)^ 3β,5α,6β-Trihydroxycholanoyl-glycine and its de-glycine metabolite are metabolites of C-triol. Accordingly, these cholenoic acids and their associated conjugates in the blood may be formed from oxysterols.

In a comparison of cholenoic acid with oxysterols for NPC biomarkers, cholenoic acids and its conjugates have advantages in terms of their simplicity of analysis (no derivatization requirements) and their sensitivity of the LC/MS/MS analysis. As a result, cholenoic acid and the conjugates are easy to analyze in LC/MS/MS. For urine, sulfuric acid conjugates were used as analytes,^45,46,81,84,97,104)^ while cholenoic acids and amino acid conjugates were analyzed in blood samples.^99,100)^

## LYSO-SPHINGOLIPIDS IN BLOOD

NPC is caused by deficiencies in the NPC1 or NPC2 proteins. However, the accumulation of sphingomyelin^1,2,105)^ and glycosphingolipids^106)^ is a characteristic of NPC. Sphingolipids accumulation is a therapeutic target of miglustat, which is an inhibitor of glucosylceramide synthase.^33)^ In 2014, Welford *et al.* reported on the LC/MS/MS analytical method for sphingosylphosphorylcholine (SPC) and glucosylsphingosine (GlcSph) and the evaluation of biomarker performance as NPC diagnostic biomarkers.^107)^ These compounds are deacylated metabolites of sphingomyelin and glucosylceramide. SPC and GlcSph have been used as biomarkers for NPA/NPB^108–111)^ and Gaucher disease,^110,112,113)^ respectively. Plasma SPC and GlcSph were analyzed using LC/MS/MS. SRM transitions were set to *m*/*z* 465>184 and 462>282 for SPC and GlcSph, respectively. Because SPC has a phosphocholine group and an amino group, it has a high ionization efficiency. GlcSph also contains an amino group that is easily ionized. A C8 column was used and a 5.5 min gradient elution was adopted for the simultaneous analysis. GlcSph contains the structural isomer galactosylsphingosine (GalSph). Although the two isomers could not be separated under reversed-phase LC conditions, the hydrophilic interaction (HILIC) LC conditions succeeded in separating them. The elution order was GlcSph and GalSph. Pretreatment was performed by solid phase extraction with OASIS HLB (Waters, Milford, MA, USA), followed by analytical method validation. The average plasma concentrations of SPC and GlcSph in patients with NPC were 2.8- and 1.4-fold-higher than those in healthy controls. The increased levels of SPC and GlcSph were lower than those of oxysterols and cholenoic acids in NPC patients. In the ROC analysis, SPC resulted in an AUC of 0.999, whereas the GlcSph AUC was 0.776. Although these sphingolipid metabolites have a lower rate of increase and lower biomarker performance than cholesterol metabolites, they are advantageous because they can be easily analyzed using LC/MS/MS without derivatization. In summary, lysosphingolipid metabolites, which are used as biomarkers for other lysosomal diseases,^108–113)^ may be considered alternative biomarkers for NPC.

## STRUCTURAL DETERMINATION AND DIAGNOSTIC PERFORMANCE EVALUATION OF *N*-PALMITOYL-*O*-PHOSPHOCHOLINE-SERINE (LYSOSPHINGOMYELIN-509) IN BLOOD

Another biomarker in the blood has also been reported. In 2015, Giese *et al.* reported on lyso-sphingomyelin-509 (lyso-SM-509) as a SPC-related metabolite.^114)^ First, the authors performed non-targeted metabolome analysis using 10 plasma samples from healthy subjects and patients with NPC to search for candidate biomarker peaks. Although the detailed method of nontargeted analysis is unknown, lyso-SM-509 was found to give rise to a NPC characteristic peak. Although our research group also employed a non-targeted lipidomics approach for other NPC blood biomarkers, lyso-SM-509 has been found to be the most characteristic biomarker for NPC.^115)^ In a report by Giese *et al.*, the chemical structure of lyso-SM-509 could not be determined.^114)^ The formula suggested from accurate mass data was C_24_H_49_N_2_O_7_P. As the difference from SPC corresponded to the mass of carbon dioxide, and the product ion detected at *m*/*z* 184 indicated the presence of a phosphorylcholine moiety, the peak denoted as lyso-SM-509. In another study,^114)^ a more detailed structural analysis was not performed. To evaluate the performance of NPC biomarkers, semi-quantitative analysis was performed using SRM analysis.^114)^ For the semi-quantification of lyso-SM-509, a calibration curve was prepared using SPC of standard solutions. The LC/MS/MS conditions reported by Welford *et al.*^107)^ were applied and the SRM transition for lyso-SM-509 was set at *m*/*z* 509>184. Lyso-globodiaosylsphingosine (lyso-Gb2) was used as an IS.^114)^ Although lyso-Gb2 is an endogenous peak, it accounts for very low concentrations in subjects, and the authors used it as an IS. The LC/MS/MS method for lyso-SM-509 was applied to the subjects. Plasma samples from patients with NPC, NPC carriers, patients with NPA/NPB, NPA/NPB carriers, and healthy subjects were analyzed. The median plasma lyso-SM-509 concentration in patients with NPC was speculated to be 6.7 ng/mL. As a result of ROC analysis, when the cutoff concentration was set at 1.4 ng/mL, the AUC value, sensitivity, and specificity were 0.99, 91%, and 100%, respectively. In addition, lyso-SM-509 was correlated with C-triol concentrations and the annual severity increment score, which is an indicator of severity.^116)^ Subsequently, the lyso-SM-509 peak was reported to be useful for NPC diagnostic screening^108,111,117)^ and was immediately cited in the guidelines.^5)^ However, these studies did not use authentic standards; therefore, chemical diagnostics based on accurate quantification were not achieved. In addition, the presence of the unknown metabolite, lyso-SM-509,^114)^ is indicative of the existence of an undiscovered pathological molecular mechanism in NPC.

**Figure 9:**
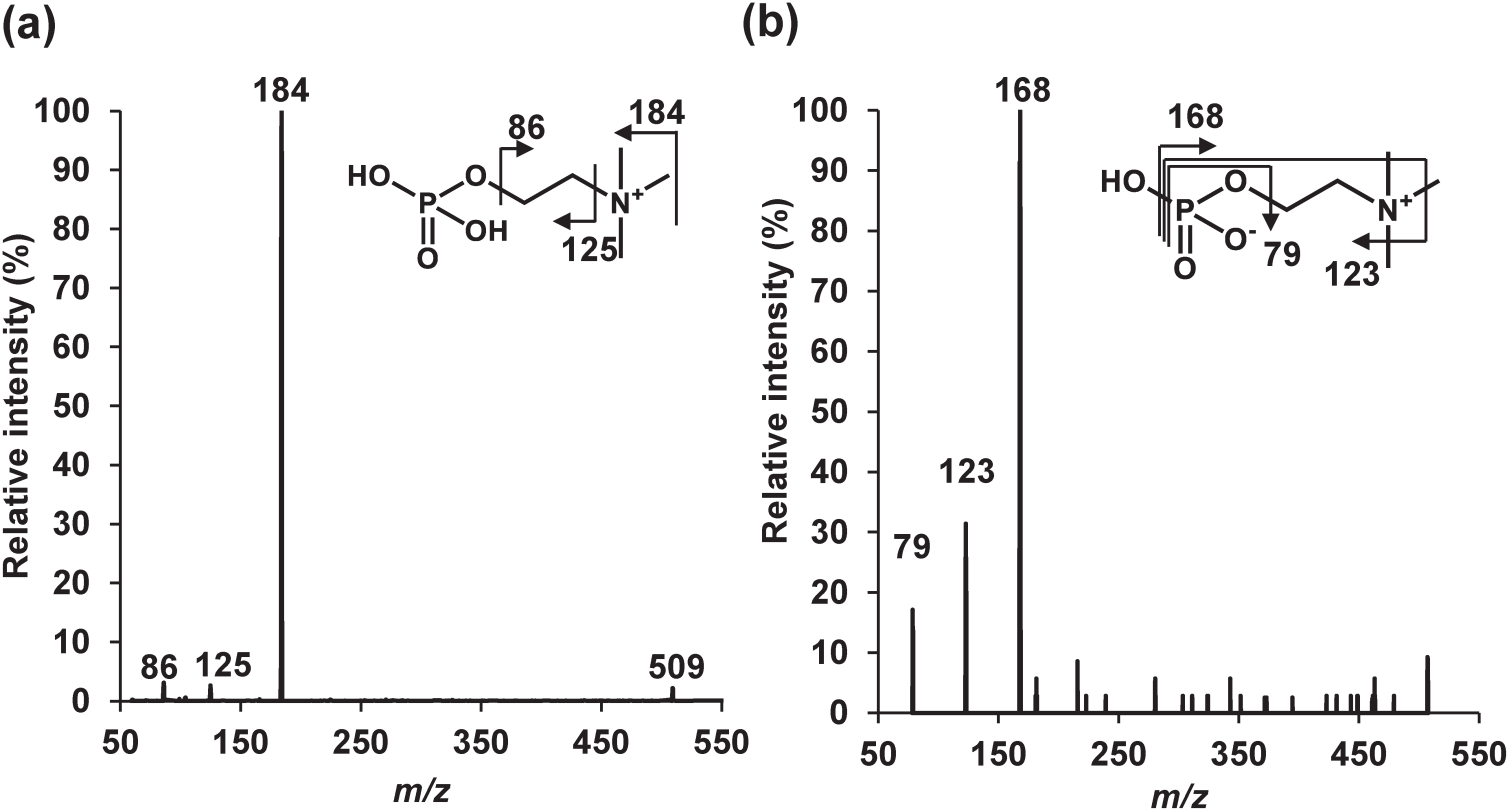
Fig. 9. Product ion spectra of serum lyso-sphingomyelin-509 under low energy CID condition. (a) positive ion mode; (b) negative ion mode.

**Figure 10:**
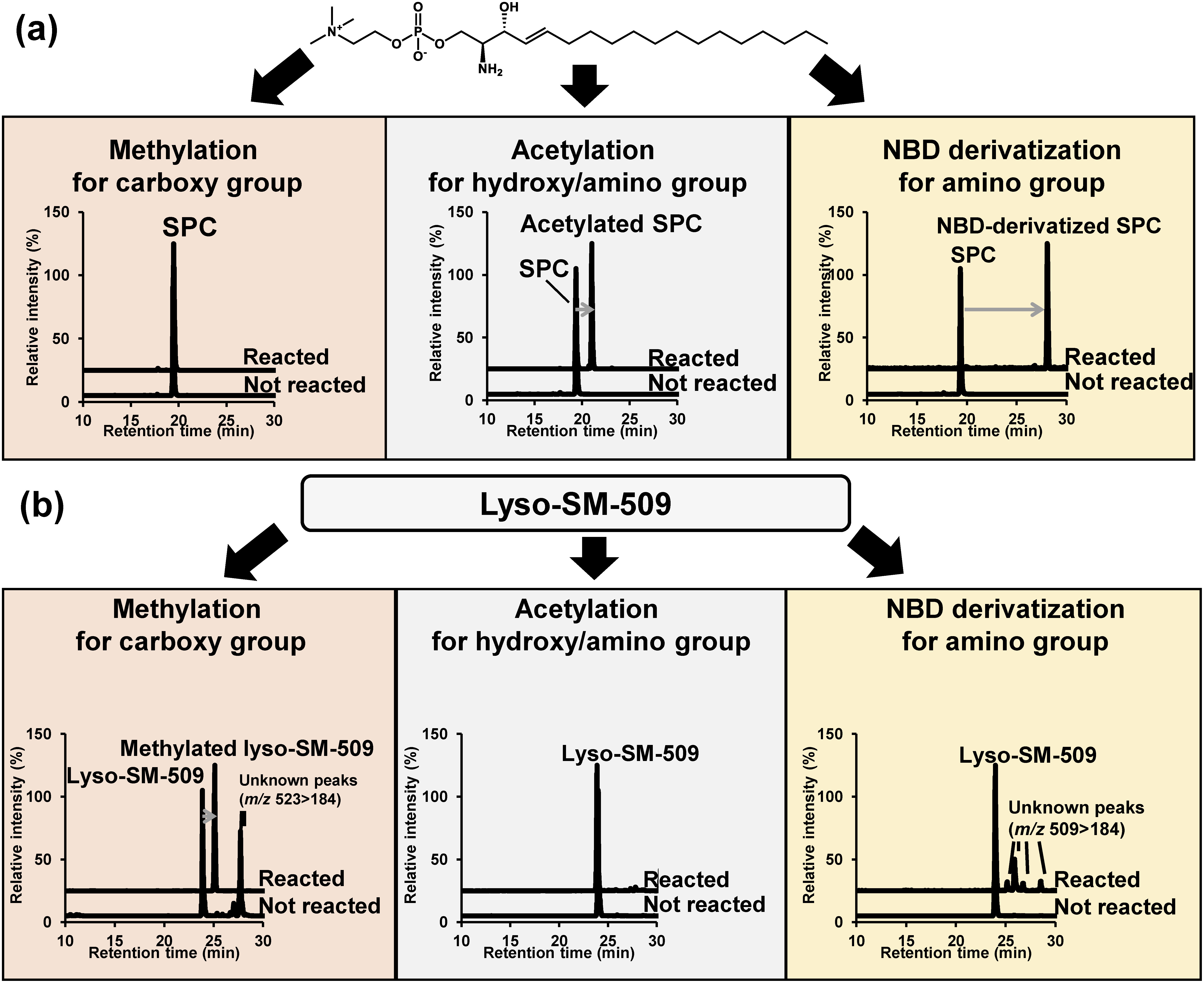
Fig. 10. Chemical derivation and LC/MS/MS analysis for structural speculation: (a) SPC; (b) lyso-SM-509.

**Figure 11:**
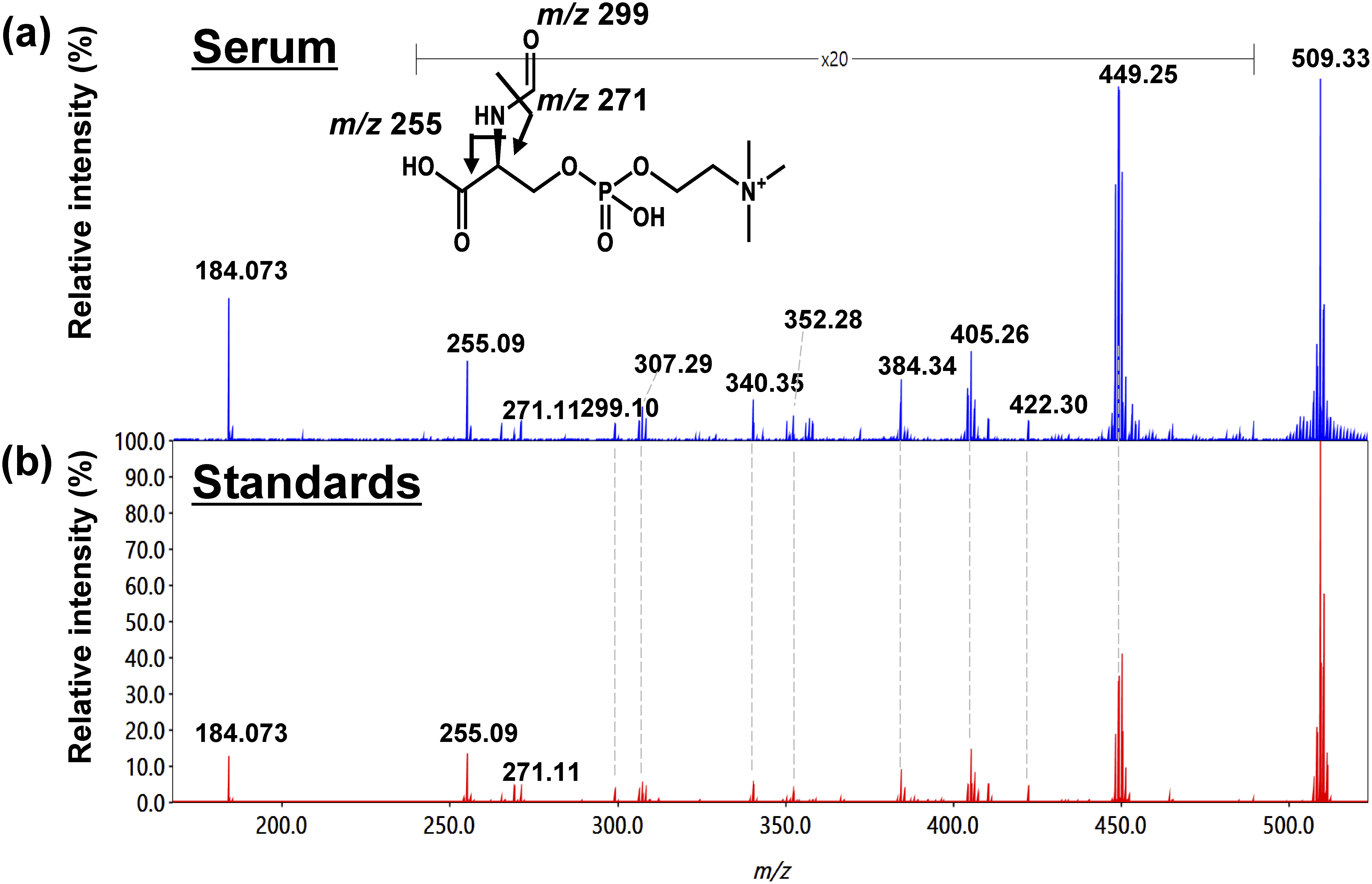
Fig. 11. Hydrogen attachment/abstraction dissociation (HAD)/MS/MS spectra: (a) serum lyso-SM-509; (b) standard.

**Figure 12:**
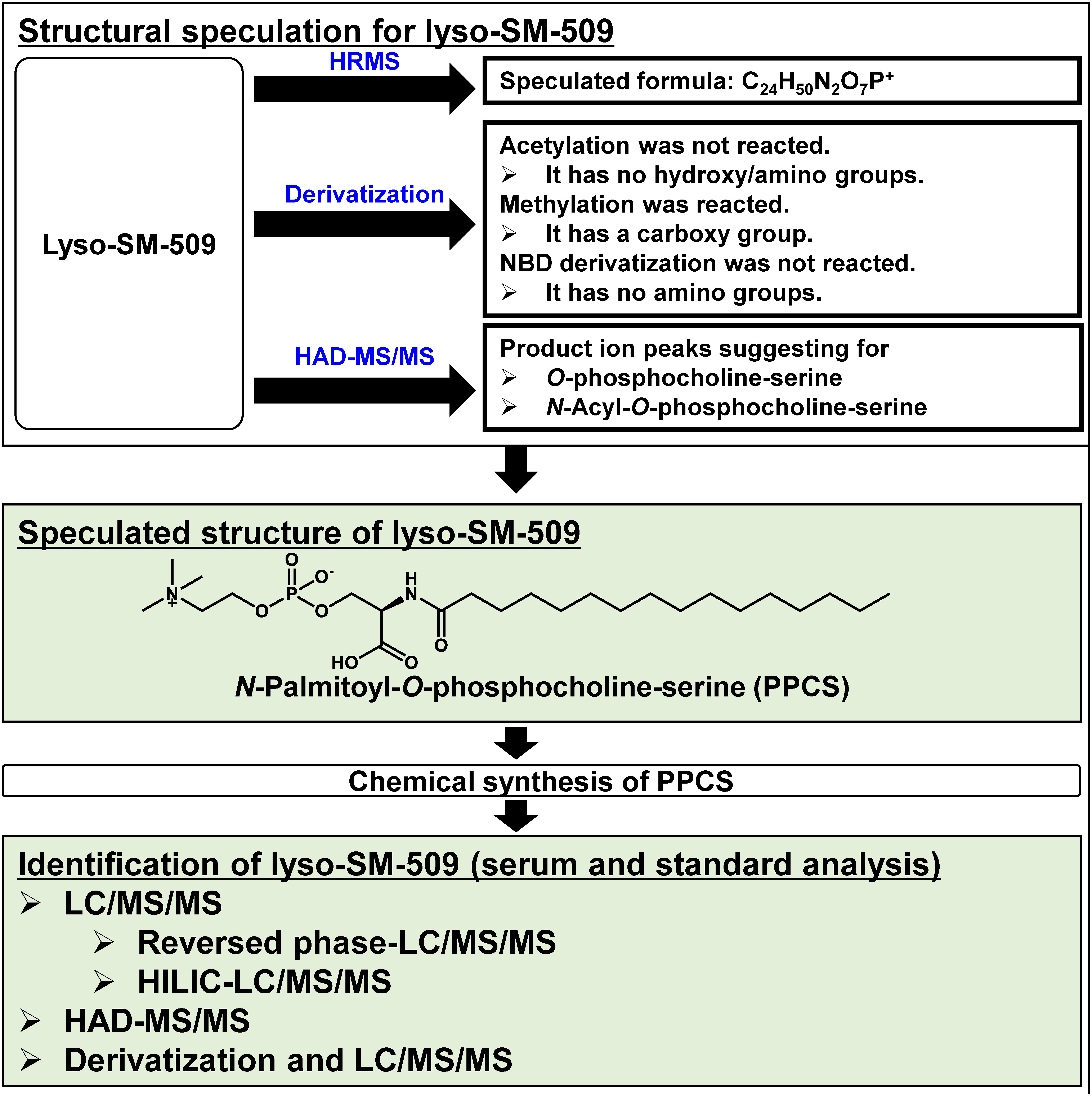
Fig. 12. Schematic representation of structural speculation and identification of PPCS. Abbreviations: HAD, hydrogen attachment/abstraction dissociation; HILIC, hydrophilic interaction; LC/MS/MS, liquid chromatography/tandem mass spectrometry; NBD, 4-fluoro-7-nitro-2,1,3-benzoxadiazol; PPCS, *N*-palmitoyl-*O*-phosphocholine-serine.

**Figure 13:**
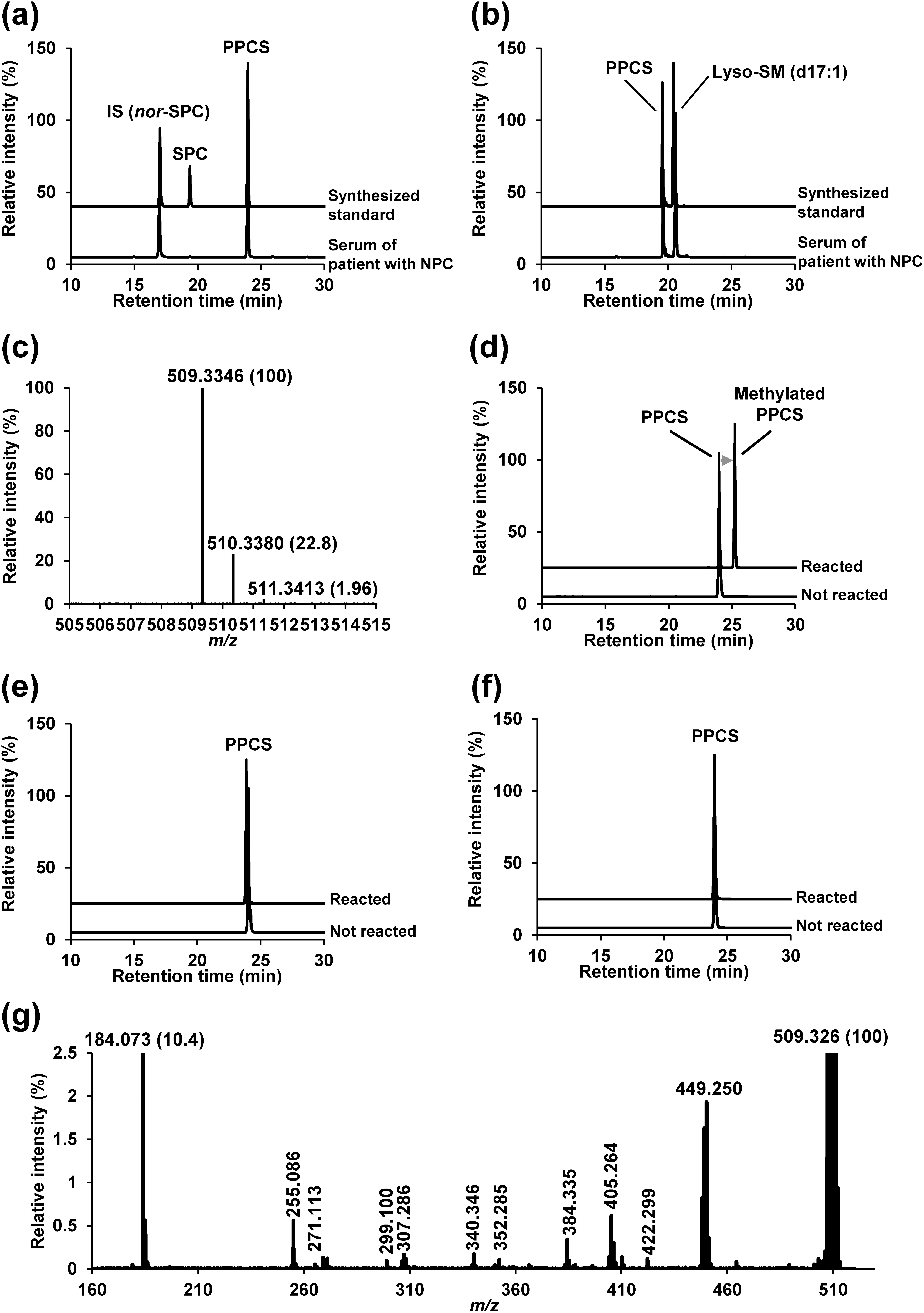
Fig. 13. Schematic representation of structural speculation and identification of PPCS: (a) Reversed-phase mode chromatographic behavior of synthesized PPCS and serum; (b) HILIC LC/MS/MS behavior of synthesized PPCS and serum; (c) typical HRMS spectrum of the synthesized PPCS; (d) SRM chromatograms of methylated PPCS (upper, reacted PPCS; lower, PPCS); (e) SRM chromatogram of acetylated PPCS (upper, reacted PPCS; lower, PPCS); (f) NBD-derivatization for PPCS (upper, reacted PPCS; lower, PPCS); (g) typical HAD MS spectrum of PPCS. Abbrevations: HAD, hydrogen attachment/abstraction dissociation; HILIC, hydrophilic interaction; NBD, 7-nitro-2,1,3-benzoxadiazole; PPCS, *N*-palmitoyl-*O*-phosphocholine-serine; SRM, selected reaction monitoring.

Subsequently, our research group aimed to determine the structure of lyso-SM-509 using various mass spectrometry techniques. Under normal low-energy CID conditions, only the product ions derived from a phosphorylcholine group were generated in both positive and negative ion modes (Fig. 9). Therefore, derivatization reactions and LC/MS/MS analyses were used to identify the functional groups. Because derivatization did not affect the phosphocholine groups, the theoretical masses of the derivatives were set as precursor ions, and all product ions were observed at *m*/*z* 184. The derivatization of serum lyso-SM-509 revealed that while the acetylation and 4-fluoro-7-nitro-2,1,3-benzoxadiazol (NBD) derivatization did not proceed, methylation did proceed. In summary, the results from lyso-SM-509 were in complete contrast to those of SPC, which is also called lyso-SM (Fig. 10). To obtain more structural information, hydrogen abstraction/attachment dissociation (HAD)-MS/MS analysis was applied.^118–120)^ This method can cleave various bonds in lipids.^119)^ The method applied product ions at *m*/*z* 299.100, 271.113, and 255.086 for serum lyso-SM-509 detection. The results from derivatization and HAD-MS/MS suggested the presence of a partial structure of *N*-acyl-phosphocholine-serine (Fig. 11). Therefore, we speculated that lyso-SM-509 was *N*-palmitoyl-*O*-phosphocholine-serine (PPCS) (Fig. 12). Next, PPCS was chemically synthesized and analyzed under the same conditions as the serum lyso-SM-509. When the LC retention times in both the reversed phase and HILIC in LC/MS/MS were compared, they were a perfect match in the serum of patients with NPC and the synthesized standard. In addition, the exact mass, HAD-MS/MS patterns, and derivatization reaction results also matched completely (Fig. 13). Therefore, we concluded that lyso-SM-509 is a PPCS.^121)^ From the simultaneous analysis of PPCS and SPC in the serum/plasma of healthy subjects, patients with NPC and other lysosomal diseases, and patients suspected of NPC without *NPC1* or *NPC2* mutations, PPCS in patients with NPC was significantly higher than that in the other subjects. In addition, a wide targeted analysis based on the *in silico* SRM conditions of theoretical masses was performed. All peaks with fatty acids other than palmitic acid (*m*/*z* 481, 537, and 565) showed higher intensity in patients with NPC than in the healthy controls. Therefore, this suggests that *N-acyl-O-phosphocholine serines* increase significantly based on NPC pathology. Sidhu *et al.* reported on the presence of these molecules at approximately the same time.^122)^ Furthermore, Sidhu *et al.* showed that the neurological disease severity score correlated with PPCS in cerebrospinal fluid. In addition, they showed that plasma PPCS decreased after the intravenous administration of HPBCD, indicating the potential of PPCS as a therapeutic biomarker for NPC.^123)^

Our research group aimed to develop a differential diagnostic method for NPC and NPA/NPB, which share common clinical symptoms. To this end, we developed two simultaneous analysis methods, a rapid analysis method and a precise simultaneous LC/MS/MS analysis method, for PPCS and SPC.^124)^ A rapid LC/MS/MS method with a run time of 1.2 min was employed using a C18 monolith short column (2.1 mm i.d. × 10 mm, 10 μm) (Monoselect C18 for HTS; GL Sciences, Tokyo, Japan), and a flow rate of LC of 1.0 mL/min. An accurate LC/MS/MS method was developed with a run time of 8.0 min using analytical method validation.^98)^ With the rapid LC/MS/MS method, the linearity of the calibration curve was high; however, the slope value was smaller than that of the accurate LC/MS/MS method. In particular, SPC concentration was significantly different between the rapid and accurate methods. In contrast, PPCS concentration was not significantly different between the rapid and accurate methods. Based on these results, we proposed a novel Niemann–Pick disease diagnostic algorithm.^124)^ All suspected subjects were screened based on their serum PPCS concentration; if the PPCS concentration was over 900 ng/mL on the rapid LC/MS/MS method, they have the potential for Niemann–Pick diseases, NPC, or NPA/NPB. Next, NPC and NPA/NPB were differentiated based on the PPCS/SPC ratio using an accurate LC/MS/MS method. If the PPCS/SPC ratio was >75, the patient was considered positive for NPC. By contrast, if the PPCS/SPC ratio was <75, they were considered positive for NPA/NPB. It has been reported that simultaneous LC/MS/MS analysis of PPCS and SPC is useful for the diagnostic screening and differentiation of NPC.^124)^

We also performed a nontargeted analysis of the serum of NPC patients and found peaks that increased or decreased in the serum of NPC patients.^115)^ Various lipids are known to be altered in NPC. With regards to the pathology of NPC, we found an increase in the peak that was speculated to be *N*-palmitoyl-serine, which has a partial structure common to PPCS. Although the metabolic pathway of PPCS is unknown, we are currently performing further analyses to gain insights into this metabolic pathway. PPCS is a lipid with a skeleton that is different from that of sphingolipids and glycerolipids (Fig. 3). With further research, it is expected that the physiological significance of these lipids and their relationship to NPC pathophysiology will be elucidated in the future.

## CONCLUSION

This review summarizes the biomarkers for NPC and the analytical methods used for their identification. MS analysis is useful for the identification, structure determination, and evaluation of diagnostic performance based on accurate quantification. In the last decade, various NPC biomarkers have been reported, all of which have been classified as lipid metabolites. In NPC, the lack of functional lysosomal cholesterol-transporting proteins results in the accumulation of cholesterol. Oxysterols, cholenoic acids, and conjugates are metabolites derived from accumulated cholesterol in NPC. SPC and GlcSph, which are lyso-metabolites of sphingolipids, have been reported as potential biomarkers, and are common in other lysosomal diseases. In addition, a novel lipid class, “PPCS,” previously known as lyso-SM-509, is an excellent biomarker for NPC. In addition, the combination of the PPCS and SPC is useful for the differential diagnosis of NPC and NPA/NPB. As PPCS and SPC have phosphocholine groups, they are sensitive and easy to measure using LC/MS/MS. In the search for NPC biomarkers, targeted, focused, and non-targeted analyses are widely used. In the structural determination of the biomarker, the use of chemical derivatization and LC/MS/MS has been effective.^121)^ To evaluate biomarkers for diagnostic performance, validated LC/MS/MS analyses are needed. Similarly, precise LC separation is useful for the separation and identification of structural isomers.^96,97)^ Biomarker analysis, and the resulting identification of novel biomarkers, will make a significant contribution to the treatment of NPC by simplifying laboratory tests for both the diagnosis and treatment of this disease. In this context, the appropriate use of chemical analyses is fundamental to the discovery of novel biomarkers and medical treatments.

## Competing Interests

The authors declare no conflict of interest.

## Author Contributions

Masamitsu Maekawa: conceptualization, writing–original draft. Nariyasu Mano: supervision.

## Abbreviations

APCI, atmospheric pressure chemical ionization; AUC, area under the curve; C-triol, 5β-cholestan-3β,5α,6β-triol; DMG, *N*,*N*-dimethylglycine; ESI, electrospray ionization; GC, gas chromatography; GlcSph, glucosylsphingosine; HAD, hydrogen attachment/abstraction dissociation; HILIC, hydrophilic interaction; HPBCD, 2-hydroxypropyl-β-cyclodextrin; HPLC, high performance liquid chromatography; HRMS, high resolution mass spectrometry; 7-KC, 7-ketocholesterol; LC, liquid chromatography; LC/MS/MS, liquid chromatography/tandem mass spectrometry; MS, mass spectrometry; MS/MS, tandem mass spectrometry; NBD, 4-fluoro-7-nitro-2,1,3-benzoxadiazol; NPA/NPB, Niemann–Pick disease types A and B; NPC, Niemann–Pick disease type C; PA, picolinic acid; PPCS, *N*-palmitoyl-*O*-phosphocholine-serine; OHC, hydroxycholesterol; S7B-Δ^5^-CA, 3β-sulfooxy-7β-hydroxy-5-cholen-24-oic acid; S7O-Δ^5^-CA, 3β-sulfooxy-7-oxo-5-cholen-24-oic acid; SNAG-Δ^5^-CA, nonamidated 3β-sulfooxy-7β-*N*-acetylglucosaminyl-5-cholen-24-oic acid; SNAG-Δ^5^-CG, glycine-amidated 3β-sulfooxy-7β-*N*-acetylglucosaminyl-5-cholen-24-oic acid; SNAG-Δ^5^-CT, taurine-amidated 3β-sulfooxy-7β-*N*-acetylglucosaminyl-5-cholen-24-oic acid; SPC, sphingosylphosphorylcholine; SRM, selected reaction monitoring; ROC, receiver operating characteristic

## Notes

Mass Spectrom (Tokyo) 2022; 11(1): A0111

Mass Spectrometry Society of Japan bestowed the MSSJ Research Award on Masamitsu Maekawa. This is an invited review of the achievement.
